# P-1780. Compliance Assessment of Antimicrobial Stewardship Program Activities within a Long Term Acute Care (LTAC) System

**DOI:** 10.1093/ofid/ofae631.1943

**Published:** 2025-01-29

**Authors:** Athena L V Hobbs, Jennifer VanCura, Katherine M Shea

**Affiliations:** Cardinal Health, Dublin, Ohio; Cardinal Health, Dublin, Ohio; Cardinal Health, Dublin, Ohio

## Abstract

**Background:**

Long Term Acute Care (LTAC) hospitals encounter a unique challenge in that despite fewer resources, they are required to comply with CMS and TJC antibiotic stewardship programs (ASPs) Conditions of Participation for hospitals. In June 2023, completion of an antimicrobial stewardship gap analysis survey was required throughout a national LTAC system to assess compliance and identify areas for improvement.

**Figure:**
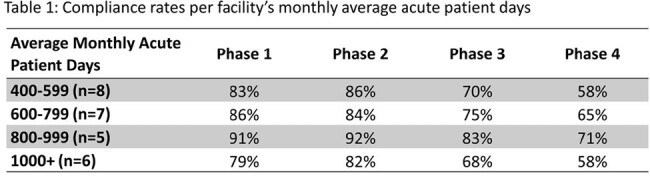

**Methods:**

This was a 173 question, multi-site survey consisting of four phases with questions about implementation of progressively more difficult ASP interventions; regulatory (n=55 questions), basic (n=45 questions), intermediate (n=41 questions), and advanced (n=32 questions) interventions. Compliance was defined as a “yes” or a “not-applicable” response. A chi-square test was used to assess responses between different phases and average monthly acute patient days (APD).

**Results:**

All 26 LTAC hospitals in the system completed the survey. The average compliance within all hospitals was 84% for phase one, 86% for phase two, 73% for phase three, and 62% for phase four. There was a significant difference in compliance between the phases (p< 0.0001) and based on APD per facility (Table 1, p< 0.0001). When assessing compliance with individual questions, a total of 13 questions (7.5%) had low compliance (less than 50%), which were flagged as particular areas for opportunity. Low compliance questions were grouped based on CDC Core Elements, six of which pertained to Action, three to Tracking, two to Education, and one each to Reporting and Hospital Leadership Commitment. As expected, the majority (n=8) of low compliance questions were in phase four.

**Conclusion:**

This standardized survey was used as a tool to ensure regulatory compliance and identify additional opportunities for ASP advancement in a system of LTACs. Overall, we report high rates of compliance, especially in the regulatory phase 1, likely due to support from a System ASP and dedicated ID pharmacist. Our evaluation suggests that smaller or less busy facilities with fewer average monthly APDs may have more resources to ensure compliance with ASP initiatives. It is important to recognize that some phase 4 advanced ASP interventions may not be feasible in the LTAC setting.

**Disclosures:**

**Katherine M. Shea, PharmD, BCIDP**, T2 Biosystems: Advisor/Consultant

